# Anti-CGRP receptor antibodies do not modulate trigeminal pain processing: indication for distinct mechanisms of CGRP pathway blockade

**DOI:** 10.1186/s10194-026-02316-z

**Published:** 2026-03-10

**Authors:** Kuan-Po Peng, Hauke Basedau, Karl Messlinger, Arne May

**Affiliations:** 1 Department of Systems Neuroscience, University Medical Center Hamburg- Eppendorf, Martinistraße 52, 20246 Hamburg, Germany; 2 Institute of Physiology and Pathophysiology, Friedrich-Alexander- Universität Erlangen-Nürnberg, Universitätsstr. 17, 91054 Erlangen, Germany

**Keywords:** QST, Sensitivity, Sensitization, Trigeminal, Preventive, Efficacy, Modulation, Switch

## Abstract

**Background:**

Monoclonal antibodies (mAbs) targeting calcitonin gene-related peptides (CGRP) are established therapies for migraine. There are currently four CGRP-mAbs available on the market: one targets the CGRP receptor, and the other three target the CGRP ligand. Despite the initial comparability of the two groups regarding efficacy, real-life data demonstrate that up to 30% of non-responders to one class exhibit a positive response to switching classes, indicating different mechanisms. The ligand-mAb, galcanezumab, has previously demonstrated a trigeminal dermatome-specific pain modulatory effect. The present study aims to evaluate the sensory modulatory effect of the receptor-mAb, erenumab.

**Methods:**

Migraine patients were recruited in two phases. In the first phase of the study, 40 patients were included and randomly assigned to receive either erenumab 70 mg (21 patients) or a placebo (19 patients) in a double-blind manner. In the second phase of the study, 46 patients were included and received erenumab 140 mg in an open-label manner. Quantitative sensory testing (QST) parameters were measured on the right forehead (V1 dermatome) and on the forearm prior to and after treatment. A repeated-measures analysis of variance (ANOVA) was used for the statistical analysis.

**Results:**

All three study cohorts (placebo, erenumab 70 mg, and erenumab 140 mg) were comparable in terms of demographics, including age, sex ratio, and baseline headache frequency, and showed no statistically significant differences in QST parameters. Subsequent to the administration of the treatment, no changes or discernible trends were observed in any of the QST parameters in any study cohort.

**Conclusions:**

The findings of this study suggest that the receptor-mAb, erenumab, did not modify the sensory thresholds following treatment. This finding is in contrast with the results of galcanezumab in the literature, which demonstrated a trigeminal sensory modulatory effect after treatment. This outcome indicates a different mechanism of action between the anti-CGRP receptor versus ligand mAbs and provides a scientific basis for the rationale of class switching, which aims to achieve additional clinical benefits in patients who are non-responders to anti-CGRP treatment.

**Preregistration:**

The study was preregistered at the Open Science Framework (https://osf.io/ygf3t).

## Background

Monoclonal antibodies (mAbs) targeting calcitonin gene-related peptides (CGRP) represent an emerging therapeutic approach in the management of both migraine and cluster headache. There are currently four CGRP-mAbs available on the market: one targets the CGRP receptor (erenumab), and the other three target the CGRP ligand [1]. During the development of these mAbs, no hypothesis was formulated that anti-ligand or anti-receptor mAbs would function in a divergent manner. It was assumed that the mechanisms of these two types of drugs should be comparable, as both act by blocking the CGRP-related signaling pathway and preventing subsequent sensitization. Initial phase 3 studies also did not demonstrate obvious disparities in clinical efficacy [2–5]. However, real-life data suggest that the two kinds of mAbs may work somewhat differently: up to 30% of non-responders to one type of mAb (e.g. receptor) may show clinical benefits upon switching to the other class [6]. Moreover, a recent functional neuroimaging study has demonstrated a class-specific central effect of ligand mAbs vs. receptor mAbs [7]. Consequently, anti-ligand and anti-receptor CGRP mAbs may exert somewhat different mechanisms of action. It is therefore hypothesized that different patient groups may benefit from different medications. Recently, it has been demonstrated that galcanezumab, an anti-CGRP-ligand mAb, exerts a trigeminal dermatome-specific pain modulatory effect, possibly by reversing existing hypersensitivity [8]. Specifically, low pain thresholds in the trigeminal dermatome increased (i.e., normalized) after treatment with galcanezumab [8]. Although these findings need to be replicated, a dermatome-specific section effect, such as that reported in the galcanezumab study, is a rather specific finding [9]. Consequently, the present study aimed to evaluate the sensory modulatory effect of the CGRP-receptor mAb – erenumab in two independent migraine cohorts.

## Methods

The study was preregistered at the Open Science Framework (https://osf.io/ygf3t) and approved by the local ethics committee of the Hamburg Chamber of Physicians (PV 5964). The study was conducted in accordance with the Declaration of Helsinki. Written informed consent was obtained before the initiation of the study.

### Study cohorts

The present study comprises two cohorts that were examined at two different times (referred to from now on as phases) of research. Phase I was a double-blind, placebo-controlled study, consisting of 44 patients who were randomly assigned to receive either erenumab 70 mg (erenumab-1) or a placebo. Phase II, as part of a multi-center EU-funded study (BioMiga), consisted of 59 patients, who received a 140 mg dose of erenumab (erenumab-2). In both phases of the study, migraine patients meeting ICHD-3 criteria aged 18–65 years were recruited from the headache outpatient clinic of the University Medical Center Hamburg-Eppendorf, Hamburg, Germany. In order to be included in the study, patients had to experience a minimum of four migraine headache days during the baseline period. The indication for erenumab therapy was determined by the updated guideline of the German Neurological Society (DGN) and the German Migraine and Headache Society (DMKG) [10]. There was no upper limit on the headache frequency for the patients recruited in Phase I. By contrast, the upper limit of headache frequency for patients recruited in Phase II was set at 25 days per month. The exclusion criteria for both cohorts were identical for the following: (1) other comorbid pain or headache disorders; (2) skin lesions on the location where quantitative sensory testing (QST) would have been performed; (3) severe psychiatric, neurologic, or other somatic comorbidities; (4) patients with additional preventative medication that was started or changed in dosage within 3 months prior to the first appointment; (5) patients with previous experience of CGRP-targeting therapy were also excluded from this study, i.e., the study cohorts were CGRP-mAb naïve. (6) For Phase I, patients with existing preventive medication who underwent a dosage change within 3 months prior to the first appointment were excluded; for Phase II, concomitant use of migraine preventive medication was not allowed. (7) The use of analgesic medication was permitted throughout the study period, with the exception of the 24-hour period preceding each appointment.

### Study protocol

**Fig. 1:**
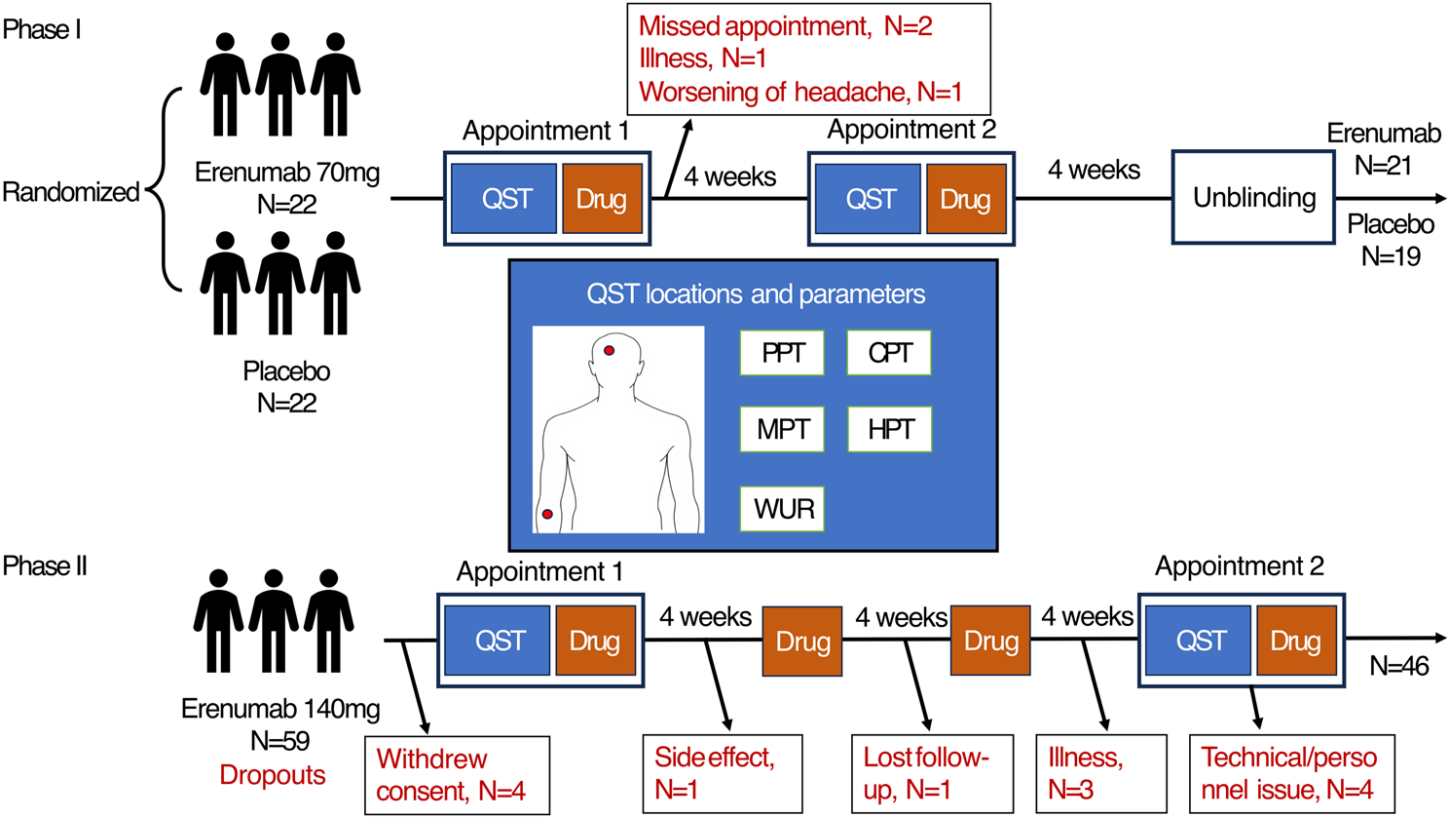
Experimental design in both phases of the study. CPT: cold pain threshold; HPT: heat pain threshold; MPT: mechanical pain threshold; PPT: pressure pain threshold; QST: quantitative sensory testing; WUR: wind-up ratio

Baseline QST parameters were measured (T0). On the day of the baseline session, following the initial measurement, a research assistant (M.S.), who was not involved in patient recruitment, data acquisition, or data analysis, administered 70 mg of erenumab or 0.9% normal saline (i.e. an equivalent amount) subcutaneously. The erenumab medication was transferred from the commercially available syringe of the pharmaceutical company to a standard syringe. This ensured that both the erenumab 70 mg and placebo injections were administered using syringes that were indistinguishable in appearance. The post-treatment QST session (T1) was scheduled following a four-week interval between sessions for the administration of the second dose of erenumab/placebo. The medication was administered subsequent to the QST session. Therefore, the post-treatment QST session occurred after one dose of either erenumab or placebo, which is comparable to the previous galcanezumab study [8]. Two months after the first dose of erenumab/placebo, all patients in Phase I were unblinded and received erenumab monthly in an open-label extension phase of the study. With the exception of the research assistant (M.S.), none of the study authors had any awareness of the treatment assignment during the data collection process. Further details including the clinical results of the double-blind (Phase I) study, have been published elsewhere [11]. In Phase II of the study, the interval between both appointments for sensory tests was three months, in accordance with the multi-centre EU-wide study protocol. In both phases, QST parameters were measured in all participants over the right forehead (V1, 2 cm above the eyebrow along the mid-pupillary line) and the right forearm (midline, 10 cm distal to the antecubital fossa). Analysis used complete case analysis, assuming data missing at random. The study protocol, including the proportion and reasons for dropout, is summarized in Fig. 1.

### Quantitative sensory testing

Standardized QST was conducted following the German Research Network on Neuropathic Pain (DFNS) protocol. Thermal sensory analyzer (TSA-II; Medoc, Ramat Yishai, Israel) was used for thermal testing; Pinprick Stimulators (7-weight, 8-512 mN; MRC Systems GmbH, Heidelberg, Germany) were used for mechanical stimuli; a nonelectrical pressure algometer, the FPK 20 (Wagner Instruments Inc, Greenwich, CT) was used for pressure pain, which has a force gauge with a 1 cm^2^ rubber tip and holds a maximum reading of 10 kg. Quantitative sensory testing parameters were measured in the following order: pressure pain threshold (PPT), mechanical pain threshold (MPT), wind-up ratio (WUR), a behavioral correlate of temporal summation for the ascending pain process which occurs at the second-order sensory neurons [12], cold pain threshold (CPT), and heat pain threshold (HPT). The selection of QST parameters was based on previous QST studies, in which a difference has been shown between different phases of migraine or between migraine patients and healthy controls [13, 14].

### Sample size justification

Given the absence of published data for erenumab on pain threshold modulation, we based our sample size calculation on the effect size observed with galcanezumab (Cohen’s d = 0.66, 95% CI 0.23, 1.08) [8]. This approach is justified by the substantial mechanistic similarity between medications: both are anti-CGRP antibodies and have been shown to reduce migraine frequency similarly [2–5]. We conservatively used d = 0.50 for the power calculation, providing a margin of safety if the effect of erenumab is smaller than that of galcanezumab. A power calculation for a matched-pairs (paired t-test) design, using a conservative Cohen’s d = 0.50, 90% power, and alpha = 0.05, yields *n* = 36 participants.

### Statistical analysis

Variables are presented as mean ± standard deviation (SD). A Kolmogorov–Smirnov test was performed to verify a normal distribution. A repeated-measures analysis of variance (ANOVA) with the Tukey post hoc test (*P* < 0.05) was performed using time (before [T0] and after erenumab [T1]) and location (V1 and forearm) as within-subject factors. The QST parameters were used as dependent variables. The Mauchly test was used to test for sphericity. When the assumption of sphericity was violated, F-values were corrected using the Greenhouse–Geisser procedure. The repeated-measures ANOVA was corrected for multiple comparisons using Bonferroni correction. All analyses were performed using Jamovi Desktop 2.3.26, an open-source statistical interface built on top of the R statistical language [15–17].

## Results

### Participant characteristics

In Phase I of the study, 44 patients were enrolled and 40 of them completed it (*n* = 19 for placebo and *n* = 21 for erenumab 70 mg). One subject in the erenumab 70 mg group withdrew due to a missed appointment, while three subjects in the placebo group withdrew due to acute illness, worsening of headaches, and claustrophobia, respectively. The overall dropout rate was 9%. In Phase II of the study, a further 59 patients were enrolled, while 46 patients completed the post-treatment follow-up appointment, resulting in a dropout rate of 22%. No significant baseline differences were identified between completers and non-completers in terms of age (41.1 ± 10.8 vs. 41.5 ± 10.8 years, *p* = 0.915), headache frequency (15.3 ± 6.0 vs. 13.7 ± 5.9, *p* = 0.400), and proportion of chronic migraine (41.7% vs. 44.4%, p=0.863). All three cohorts (placebo, erenumab-1, and erenumab-2) were comparable in terms of age, sex ratio, and baseline headache frequency. The complete clinical features are summarized in Table 1. For the placebo, erenumab-1, and erenumab-2 groups, the 50% responder rate (headache frequency reduction ≥ 50%) was 5.3%, 19.1%, and 47.8%, respectively, and the 30% responder rate (headache frequency reduction ≥ 30%) was 21.1%, 33.3%, and 71.7% respectively.

**Table 1:** Description of the study cohorts

	Phase I		Phase II	*P*-value
	Cohort 1 (placebo)	Cohort 2 (erenumab-1)	Cohort 3 (erenumab-2)	
Case number	19	21	46	
Sex (female, %)	15 (78.9%)	19 (90.5%)	41 (89.0%)	0.495^1^
Age (mean ± SD)	41.6 ± 11.4	39.1 ± 12.8	41.1 ± 12.0	0.789^2^
Migraine with aura	10 (52.6%)	8 (38.1%)	N/A	
Headache frequency at baseline (days/month)	13.3 ± 6.5	15.7 ± 8.3	15.3 ± 6.0	0.384^3^
Chronic migraine (%)	21.1%	42.9%	45.7%	0.171^1^
Concomitant use of other preventive medications	6 (31.5%)	6 (28.5%)	0 (0%)	< 0.001^1^

^1^Chi-Square/Fisher’s exact test; ^2^One-Way ANOVA; ^3^Kruskal-Wallis Test

### Effects of erenumab on quantitative sensory tests

The baseline QST parameters were comparable among the three study cohorts. The proportion of patients who reported having experienced headaches on the day of examination is as follows: The percentages obtained for the first session were 42.1%, 52.3% and 56.5%, respectively, for the placebo, erenumab-1, and erenumab-2 cohorts. For the second session, the percentages obtained were 36.8%, 42.9% and 26.1%, respectively. Subsequent to the administration of treatment, no alterations or discernible trends were observed in any of the QST parameters following erenumab or placebo treatment in any of the three study cohorts (placebo, erenumab-1, and erenumab-2). The effect sizes were consistently small for the QST parameters that had been previously reported to be increased after treatment with galcanezumab [8], i.e., HPT and MPT in the V1 dermatome (|Cohen’s d| < 0.20 in all three cohorts). The details of the comparison of QST parameters and the statistical analyses are summarized in Table 2.

**Table 2:** Effecerst of erenumab on QST paramet

QST parameter	Cohort 1 (placebo)				Cohort 2 (erenumab-1)				Cohort 3 (erenumab-2)			
	Before/ After	Mean difference [95% CI]	Cohen’s d [95% CI]	*P*-value	Before/ After	Mean difference [95% CI]	Cohen’s d (95% CI)	*P*-value	Before/ After	Mean difference [95% CI]	Cohen’s d (95% CI)	*P*-value
**V1 Dermatome**												
Heat pain threshold (°C)	43.8 ± 0.8/ 43.9 ± 0.7	0.05 [-1.40,1.50]	-0.02 [-0.47,0.43]	0.940	43.2 ± 0.8/ 43.6 ± 0.8	0.34 [-1.59, 2.27]	-0.08 [-0.51,0.35]	0.715	43.0 ± 0.6/ 43.1 ± 0.6	0.08 [-1.14,1.30]	-0.02 [-0.31,0.27]	0.899
Cold pain threshold (°C)	13.1 ± 2.0/ 12.7 ± 1.5	-0.43 [-4.1,3.1]	0.06 [-0.39,0.50]	0.805	13.6 ± 1.7/ 14.1 ± 1.8	0.45 [-2.87,3.77]	-0.06 [-0.49,0.37]	0.780	15.1 ± 1.3/ 15.9 ± 1.1	0.82 [-1.76,3.39]	-0.09 [-0.38,0.20]	0.527
Mechanical pain threshold (log mN)	1.58 ± 0.07/ 1.50 ± 0.08	0.09 [-0.25,0.11]	0.19 [-0.26,0.65]	0.396	1.35 ± 0.06/ 1.37 ± 0.07	0.02 [-0.09,0.13]	-0.10 [-0.52,0.33]	0.666	1.34 ± 0.05/ 1.36 ± 0.05	0.02 [-0.08,0.12]	-0.06 [-0.35,0.23]	0.678
Pressure pain threshold (log kPa)	2.43 ± 0.04/ 2.43 ± 0.03	0.00 [-0.08,0.08]	0.00 [-0.45,0.45]	0.998	2.51 ± 0.04/ 2.46 ± 0.04	-0.05 [-0.12,0.02]	0.31 [-0.13,0.75]	0.168	2.46 ± 0.02/ 2.43 ± 0.02	-0.04 [-0.08,0.01]	0.21 [-0.08,0.51]	0.150
Wind up ratio	3.21 ± 2.44/ 3.17 ± 2.58	-0.04 [-1.10,1.02]	0.02 [-0.43,0.47]	0.934	4.29 ± 2.58/ 4.65 ± 4.95	0.36 [-1.44,2.15]	-0.09 [-0.52,0.34]	0.684	3.77 ± 3.43/ 3.47 ± 2.45	-0.31[-1.32,0.70]	0.08 [-0.20,0.38]	0.545
**Forearm**												
Heat pain threshold (°C)	42.8 ± 0.8/ 42.6 ± 0.8	-0.27 [-2.11,1.56]	0.07 [-0.38,0.52]	0.758	43.8 ± 0.8/ 43.9 ± 0.8	0.14 [-1.75,2.03]	-0.03 [-0.46,0.39]	0.878	43.1 ± 0.5/ 43.7 ± 0.5	0.61 [-0.32,1.53]	-0.20 [-0.49,0.09]	0.191
Cold pain threshold (°C)	11.7 ± 1.9/ 14.5 ± 1.8	2.81 [-0.81,6.42]	-0.37 [-0.84,0.09]	0.120	13.8 ± 1.8/ 14.3 ± 2.0	0.56 [-2.63,3.74]	-0.08 [-0.51,0.35]	0.718	12.1 ± 1.5/ 12.0 ± 1.2	-0.06 [-3.57,3.45]	0.00 [-0.28,0.29]	0.974
Mechanical pain threshold (log mN)	1.18 ± 0.08/ 1.13 ± 0.08	-0.05 [-0.19,0.09]	0.18 [-0.28,0.63]	0.455	1.16 ± 0.05/ 1.16 ± 0.04	0.01 [-0.08,0.09]	-0.03 [-0.46,0.40]	0.892	1.09 ± 0.04/ 1.08 ± 0.04	-0.01 [-0.09,0.07]	0.04 [-0.25,0.33]	0.797
Pressure pain threshold (log kPa)	2.49 ± 0.18/ 2.48 ± 0.11	-0.01 [-0.08,0.06]	0.07 [-0.38,0.52]	0.774	2.52 ± 0.03/ 2.47 ± 0.04	-0.05 [-0.13,0.02]	0.32 [-0.12,0.76]	0.156	2.52 ± 0.03/ 2.48 ± 0.03	-0.04 [-0.10,0.01]	0.23 [-0.06,0.52]	0.125
Wind up ratio	3.99 ± 3.67/ 3.16 ± 2.39	-0.84 [-2.55,0.99]	0.23 [-0.22,0.69]	0.320	3.86 ± 2.50/ 4.29 ± 3.55	0.43 [-1.04,1.90]	-0.13 [-0.56,0.30]	0.551	3.79 ± 3.31/ 3.47 ± 2.85	-0.31 [-1.32,0.69]	0.09 [-0.20,0.38]	0.533

QST: quantitative sensory testing; V1: first branch of the trigeminal dermatome, QST data are presented with mean ± SE

## Discussion

Placebo demonstrated no modulatory effect on sensory threshold in either a trigeminal or an extracranial dermatome. However, in contrast to the hypothesis and to the findings in a previous galcanezumab study [8], erenumab did not exert any modulatory effect on sensory threshold either. Notably, CGRP has been shown to induce sensitization in humans. The absence of any modulatory effect resulting from blocking of CGRP receptors suggests the existence of an alternative mechanism in CGRP-induced sensitization, beyond the binding between the CGRP and the CGRP-receptor. Moreover, as the cohort characteristics of the two erenumab cohorts were similar to the historical galcanezumab cohort in terms of age, sex ratio, and baseline proportion of chronic versus episodic migraine [8], the data imply that erenumab, as opposed to galcanezumab, did not alter the sensory threshold after treatment. Further confirmation is required in the form of a direct head-to-head comparison study between the two types of CGRP-mAbs. Nevertheless, the difference in pain threshold modulation provides the rationale for the transition from an anti-CGRP-receptor antibody to an anti-CGRP-ligand antibody [6, 18]. As was also noted in recent neuroimaging studies [7, 11, 19], the mechanisms of action between the ligand and receptor antibodies may be considerably different.

Clinical evidence has already suggested that antibodies that target either CGRP ligands or receptors may function somewhat differently. Approximately 30% of the patients who do not respond to the anti-CGRP-receptor mAb – erenumab, may still benefit from a shift to an anti-CGRP-ligand mAb [20, 21]. Moreover, the transition from an anti-CGRP-ligand antibody to an anti-CGRP-receptor antibody also exhibits a comparable level of efficacy in initial nonresponders [22]. The most salient conclusion that can be drawn from these studies is that the two types of antibodies may function in a somewhat divergent manner, rather than one type being demonstrably superior to the other. Another peptide associated with the pathophysiology of migraine, pituitary adenylate cyclase-activating peptide (PACAP), exhibited divergent outcomes from anti-ligand and anti-receptor treatment modalities. The clinical trial of the anti-PACAP-receptor – PAC1 antibody, revealed no therapeutic benefit over placebo for migraine prevention [23]. However, the phase 2 study investigating an anti-PACAP-ligand antibody for migraine prevention was successful and has been recently published [24]. The efficacy of the anti-ligand, but not the anti-receptor antibody targeting PACAP, further indicates that the blocking of the receptors and ligands may yield divergent outcomes, presumably due to disparate mechanisms. One theory that is specific to the anti-ligand, as opposed to the anti-receptor mAb, is based on the CGRP-sink theory – CGRP released from the nerve fibers innervating the pial blood vessels becomes concentrated in the cerebrospinal fluid (CSF) due to its inability to effectively traverse the blood-brain barrier (BBB), thereby preventing it from degradation in the periphery [25]. There is a broad consensus that CGRP-mAbs demonstrate minimal permeability across the BBB, with a reported permeability of less than 0.1%. This finding has been attributed to their molecular size [26]. However, a recent study found that the CSF-to-plasma ratio of fremanezumab can reach up to 0.36% [27]. This amount may be sufficient to bind all the CGRP in the CSF [25]. Conversely, CGRP-receptor antibodies must be able to reach specific target regions in the brain that are relevant to migraine pathophysiology to exert their efficacy.

The mechanisms that underlie the different pain threshold modulatory effects between anti-ligand and anti-receptor mAbs remain speculative in the present study. There are at least two possible mechanisms involved. Evidence from neuroimaging research suggests differential effects of ligand- and receptor-targeting mAbs at the central level. A direct comparison of galcanezumab and erenumab revealed differential effects on the hypothalamus and thalamus [7]. A preclinical study has demonstrated that fremanezumab, another anti-CGRP-ligand mAb, reduced the facial sensitivity to noxious mechanical and thermal stimulation in rodents [28]. The study utilized a behavioral measurement of avoidance incorporating tolerability and motivation as a means to assess pain sensitivity. As the behavioral measurement involves complex central mechanisms, the authors conclude that their findings must involve a central component, rather than purely peripheral ligand blocking [28].

In addition to a central mechanism, the differences between erenumab and galcanezumab can also be explained at the peripheral level. CGRP levels in the external jugular vein, saliva, and tears have been shown to increase during spontaneous migraine attacks [29–31]. However, the evidence for an increase in CGRP level in a non-trigeminal dermatome during a migraine attack is either non-existent or controversial [32], suggesting that the role of CGRP in migraine is trigeminal-specific. Notably, the subcutaneous injection of CGRP does not induce pain directly [33]. Instead, it decreases the pain threshold (and therefore induces sensitization) [34]. It has been suspected that galcanezumab exerts its pain-modulatory effect by reversing the CGRP-induced hypersensitivity [8]. One hypothesis to explain why only an anti-CGRP-ligand antibody, but not an anti-CGRP-receptor antibody, can reverse CGRP-induced sensitivity involves an alternative receptor. Given that CGRP not only binds to CGRP receptors, but also to the amylin-1 receptor, with a binding affinity similar to that of amylin [35], it can be hypothesized that the binding between CGRP and the amylin-1 receptor is sufficient to maintain the high-sensitivity state in the trigeminal system. In animal models, the injection of amylin has been demonstrated to induce mechanical hyperalgesia [36], a phenomenon analogous to that observed with the injection of CGRP [37, 38]. However, the blocking of CGRP receptor alone, as opposed to blocking of the CGRP ligand, did not prevent the binding of CGRP to the amylin-1 receptor, and thus no modulatory effect may be observed in erenumab in terms of trigeminal nociceptive sensitivity. The validity of these theories is yet to be substantiated through studies that measure the mechanism directly.

### Limitations

Firstly, it could be argued that the sample size of the first erenumab cohort (*n* = 21) did not reach the expected number of 36 subjects, as determined by the sample size calculation. Consequently, we recruited a second and independent cohort (*n* = 46). This showed very similar results without even a trend towards a difference. Secondly, the participants in Phase I and II were comparable in terms of age, baseline headache frequency, and proportion of chronic migraine. However, the differences in study design (double-blind vs. open-label), the dosage difference of erenumab, and the different interval between the first and second appointments render a direct comparison between the two phases difficult and the pooling of the results not feasible. Thirdly, QST parameters have been demonstrated to be operator-dependent and have been shown to exhibit significant interpersonal variation [39]. In the present study, both these factors were well controlled. This was achieved by employing a longitudinal design, whereby each patient served as his or her own control. Furthermore, over 90% of the QST measurements were conducted exclusively by the same operator (K.P.), thereby precluding the possibility of inter-operator variability. Notwithstanding the absence of significant findings, some QST parameters demonstrated effect sizes above 0.5 within the 95% confidence interval. This suggests a degree of uncertainty that requires confirmation in larger samples. Finally, the data from the galcanezumab study were, despite being comparable in every aspect, from a historical control. Consequently, the data do not permit a causal relationship that would indicate that anti-ligand vs. anti-receptor drugs are the sole mechanism responsible for the observed differences in terms of pain threshold modulation. The optimal approach would be the execution of a randomized parallel study, with the objective of elucidating the discrepancy between anti-CGRP-ligand and -receptor antibodies. The question of whether the observed difference in the modulation of trigeminal nociception in the present study can be translated into a clinically meaningful difference remains to be investigated.

## Conclusions

The present study provides new evidence suggesting a possible different modulatory effect on trigeminal nociception between an anti-CGRP-ligand and an anti-CGRP-receptor antibody. This establishes the basis for the potential benefits of class switching for patients who have demonstrated an unsatisfactory response to erenumab. How the difference in sensory modulatory effect translates to a difference in clinical efficacy is worthy of further investigation.

## Data Availability

Researchers meeting the criteria for access to confidential data may access the data upon reasonable request, including the documentation of data access.
